# Driver’s licence in an adult population with good vision: an exploratory study in two longitudinal very low birthweight cohorts

**DOI:** 10.1136/bmjophth-2025-002451

**Published:** 2026-01-14

**Authors:** Tora Sund Morken, Dordi Austeng, Maarit Kulmala, Eero Kajantie, Kari Anne Indredavik Evensen, Anna Majander

**Affiliations:** 1 Department of Neuromedicine and Movement Science, Norwegian University of Science and Technology, Trondheim, Norway; 2 Department of Ophthalmology, St. Olav hospital, Trondheim University Hospital, Trondheim, Norway; 3 Welfare Epidemiology and Monitoring Unit, Finnish Institute for Health and Welfare, Helsinki, Finland; 4 Department of Ophthalmology, Helsinki University Hospital, Helsinki, Finland; 5 Clinical Medicine Research Unit, University of Oulu, Oulu, Finland; 6 Department of Clinical and Molecular Medicine, Norwegian University of Science and Technology, Trondheim, Trøndelag, Norway; 7 Children’s Clinic, St. Olav hospital, Trondheim University Hospital, Trondheim, Norway; 8 Department of Rehabilitation Science and Health Technology, Oslo Metropolitan University, Oslo, Norway

**Keywords:** Child health (paediatrics), Epidemiology

## Abstract

**Objective:**

To investigate whether being born preterm with very low birth weight (VLBW), lower best-corrected visual acuity (BCVA) or a history of a psychiatric or somatic diagnosis is associated with, or mediates, not having obtained a driver’s licence at adult age.

**Methods and analysis:**

Potential predictors of no driver’s licence were investigated in participants with a mean age of 36 years and BCVA above the legal limit to drive from the Norwegian University of Science and Technology Low Birth Weight Life Study, Norway, and the Helsinki Study of Very Low Birth Weight Adults, Finland, (VLBW n=119, term-born controls n=149).

**Results:**

Participants with no driver’s licence (n=34) had lower BCVA than participants with a driver’s licence (mean logarithm of the minimal angle of resolution (SD) −0.03 (0.11) vs −0.101 (0.09), p<0.001. Being born with VLBW and lower BCVA was associated with no driver’s licence (OR, 2.5 (95% CI 1.1 to 5.8) and 1.1 per unit (95% CI 1.0 to 1.2), respectively. BCVA was not a mediator of the effect of being born preterm with VLBW.

**Conclusion:**

In a population with good visual acuity, being born preterm with VLBW was a predictor for not having a driver’s licence, while lower visual acuity further increased this likelihood. A history of a somatic or psychiatric diagnosis was not associated. These findings should be interpreted with caution due to the relatively small sample size and a non-negligible statistical uncertainty. The causal relationship between being born preterm and not holding a driver’s licence remains to be investigated.

**WHAT IS ALREADY KNOWN ON THIS TOPIC**

- The proportion of adults born preterm with very low birth weight (VLBW) that does not drive is larger than that of term-born peers, but the cause is unknown.

**WHAT THIS STUDY ADDS**

- In a population with visual acuity above the legal limit to drive, being born preterm with VLBW and lower visual acuity was associated with not having obtained a driver’s licence.

**HOW THIS STUDY MIGHT AFFECT RESEARCH, PRACTICE OR POLICY**

- These findings may indicate that brain function is involved, but the causal relationship between being born preterm and not holding a driver’s licence remains to be investigated.

## Introduction

 Successfully obtaining a driver’s licence depends on multiple factors including the general health, intellectual capacity and/or sensory function of an individual. The proportion of adults born preterm with very low birth weight (VLBW) who have not obtained a driver’s licence^1^ and the proportion that does not drive^2 3^ is larger than that of term-born peers. Furthermore, VLBW adults have a lower best-corrected visual acuity (BCVA) than controls.^1^ Moreover, the prevalence of mental health disorders is higher in individuals born preterm with VLBW than in term-born peers.^4^ This is also true for a wide range of somatic disorders.^4 5^ A psychiatric or somatic medical history may potentially be indicators of health-related obstacles to obtaining a driver’s licence, and it is of interest to explore potential factors that may be associated with this. To this end, it was investigated whether being born preterm with VLBW, lower BCVA or a history of a psychiatric or somatic diagnosis is associated with, or mediates, not having obtained a driver’s license at adult age.

## Methods

Data were collected from the Norwegian University of Science and Technology Low Birth Weight in a Lifetime Perspective Study (NTNU LBW Life) and the Helsinki Study of Very Low Birth Weight Adults (HeSVA) during 2019–2020.^1 6^ The study population was restricted to those with BCVA≥0.3 logarithm of the minimal angle of resolution (logMAR) in at least one eye (VLBW n=124; control n=149) as this is the legal limit for obtaining a driving licence. The NTNU LBW Life is a hospital-defined cohort where VLBW infants admitted to St. Olav’s Hospital, Trondheim, Norway during 1986–1988 were recruited. Term-born controls were born >gestational week 37 with a birth weight>10th percentile for gestational age.^7^ The HeSVA is a geographically defined birth cohort where infants discharged alive from the neonatal intensive care unit of the province of Uusimaa, Finland during 1978–1985 were recruited. For each VLBW, a same-sex not small for gestational age term-born infant was recruited as a control. Background data were collected from medical records (table 1). BCVA was obtained at 4 m according to the Early Treatment Diabetic Retinopathy Study protocol.^8^ The participants were asked to report whether they had been diagnosed (by a physician) with one of the following conditions: attention deficit hyperactivity disorder, autism, bipolar disorder, depression, panic, social and general anxiety disorder, obsessive compulsive and post-traumatic stress disorder, schizophrenia or other psychosis, cancer, cerebral palsy, chronic fatigue, epilepsy, diabetes mellitus type I and II, high blood pressure, heart disease, hypothyroidism and hyperthyroidism, intestinal disease, osteoporosis, polycystic ovary syndrome, rheumatoid disease and stroke.

**Table 1:** Background characteristics of the VLBW and control group in the HeSVA and the NTNU LBW Life

	VLBW	Control
	n=119	n=149
GA (weeks)	29.6 (2.4)	40.0 (1.2)
Birth weight (g)	1184 (207)	3647 (482)
BPD n (%)	28 (24)	0 (0)
Sepsis n (%)	8 (7)	0 (0)
Supplemental oxygen days	19 (40)	1 (–)
HeSVA n (%)	82 (73)	88 (59)
NTNU n (%)	37 (31)	61 (41)
Age at follow-up (years)	36 (3)	36 (3)
Female n (%)	70 (58)	87 (58)
NSI, n (%)	9 (8)	1 (1)
BCVA (letters)	88.9 (5.2)	90.2 (4.4)
No driver’s licence n (%)	24 (20)	10 (7)

The numbers are mean (SD) or n (%). BPD is defined as need for supplemental oxygen≥28 days. NSI is defined as cerebral palsy, blindness, deafness (HeSVA) or hearing aid (NTNU), intellectual disability (HeSVA) or IQ<70 at 19, 14, 5 years of age (NTNU).

BCVA, best-corrected visual acuity; BPD, bronchopulmonary dysplasia; GA, gestational age; HeSVA, Helsinki Study of Very Low Birth Weight Adults; NSI, neurosensory impairment; NTNU LBW Life, Norwegian University of Science and Technology Low Birth Weight in a Lifetime Perspective Study.

### Statistical analyses

In the first step, it was investigated if participants with no driver’s licence had a lower BCVA than participants with driver’s licence. To identify potential predictors, the proportion of participants with and without a driver’s licence was cross-tabulated with the number of participants in either the VLBW or in the control group. Following this, the proportion of participants with and without a driver’s licence was cross-tabulated with self-reported ever having had a psychiatric/somatic diagnosis or not. For comparisons, an independent samples t-test was applied for continuous (BCVA) and two-sided χ^2^ test for categorical variables. Logistic regression was performed to assess the impact of these variables on the likelihood of no driver’s licence. The multiple logistic regression model contained four independent variables (VLBW, ever a somatic or psychiatric diagnosis, BCVA) adjusted for sex, age at follow-up and cohort. ORs for no driver’s licence with 95% CIs were reported. Mediation analyses were performed to investigate the potential causal pathway from VLBW to no driver’s licence with BCVA as a mediator. Effects from mediation analyses are reported as risk differences adjusted for cohort, age and sex. A p value<0.05 was considered statistically significant. Statistical analyses were performed using SPSS software (V.30.0) and Stata (mediation analyses; V.18.0, StataCorp, Texas, USA).

### Patient and public involvement

Patients or the public were not involved in the design, conduct, reporting or dissemination plans of our research.

## Results

Data on both driver’s licence and BCVA were available for 119 VLBWs and 148 control participants. 20% (n=24) of the VLBWs and 7% (n=10) of the control participants had no driver’s licence. Mean (SD) BCVA was −0.03 (0.11) logMAR in the no driver’s licence group versus −0.101 (0.09) logMAR in the driver’s licence group, p<0.001. Participants with no driver’s licence were more often VLBW and more often had a history of a somatic or psychiatric diagnosis (table 2).

**Table 2:** Predictors of not having a driver’s licence among participants with BCVA≥70 ETDRS letters in at least one eye in the NTNU LBW Life Study and the Helsinki Study of Very Low Birth Weight Adults

	No driver’s licence	Driver’s licence	P value
	n=34	n=233	
BCVA (logMAR)	−0.03 (0.11)	−0.101 (0.09)	<0.001
Very low birth weight n (%)	24 (71)	95 (41)	0.001
Somatic diagnosis n (%)	17 (50)	63 (27)	0.006
Psychiatric diagnosis n (%)	16 (47)	56 (24)	0.005

The numbers are mean (SD) or n (%). Driver’s licence is defined as answer yes to the question ‘Do you have a driver’s licence?’ Somatic diagnosis is defined as a self-reported history of a somatic diagnosis diagnosed or treated by a physician. Psychiatric diagnosis is defined as a self-reported history of a psychiatric diagnosis diagnosed or treated by a physician.

BCVA, Best-corrected visual acuity; ETDRS, Early Treatment Diabetic Retinopathy Study; logMAR, logarithm of the minimal angle of resolution; NTNU LBW Life Study, Norwegian University of Science and Technology Low Birth Weight in a Lifetime Perspective Study; VLBW, Very Low Birth Weight.

### Multiple logistic regression and mediation analyses

Being born VLBW and lower BCVA were significant predictors for no driver’s licence with OR of 2.5 (95% CI 1.1 to 5.8) and 1.1 (95% CI 1.0 to 1.2; table 3). Lower BCVA was not a mediator of the effect of being born VLBW on no driver’s licence at adult age (table 4).

**Table 3:** Predictors associated with not having a driver’s licence in participants with BCVA≥70 ETDRS letters in at least one eye in the NTNU LBW Life Study and the Helsinki Study of Very Low Birth Weight Adults

Covariates	OR	95% CI	P value
Univariate analyses			
VLBW	3.6	1.6 to 8.0	0.002
Multivariate analyses			
VLBW	2.5	1.1 to 5.8	0.034
Somatic diagnosis	2.1	0.9 to 4.6	0.076
Psychiatric diagnosis	2.2	1.0 to 5.0	0.053
BCVA in the best eye per unit	1.1	1.0 to 1.2	0.003

All analyses are adjusted for age, sex and cohort. Somatic or psychiatric diagnoses were defined as a history of somatic or psychiatric diagnosis ever diagnosed by a clinician, reported by the participant.

ETDRS, Early Treatment Diabetic Retinopathy Study; HeSVA, Helsinki Study of Very Low Birth Weight Adults; NTNU LBW Life, Norwegian University of Science and Technology Low Birth Weight in a Lifetime Perspective Study; VLBW, very low birth weight.

**Table 4:** Effect given as risk differences of VLBW versus control on not having a driver’s licence with BCVA as a mediator adjusted for cohort, age and sex

	n*	Direct effect of VLBW	Indirect effect of VLBW	Total effect of VLBW
No driver’s licence	119, 148	0.10 (0.02 to 0.18)	0.02 (−0.004 to 0.05)	0.13 (0.04 to 0.2)

Results are given as risk difference estimates with 95% CI. Mediation analyses were performed to investigate the potential causal pathway from VLBW to no driver’s licence (total effect of VLBW) with BCVA as a mediator (indirect effect of VLBW). Effects from mediation analyses are reported as risk differences adjusted for cohort, age and sex.

* VLBW, control.

BCVA, best-corrected visual acuity; VLBW, very low birth weight.

### Post hoc power analyses and size of the type 2 error

With a sample of 34 participants with no driver’s licence and 233 with a driver’s licence the study has a power of 76.4 and 78.3%, respectively, to answer whether the distribution of a history of a somatic or psychiatric diagnosis differs between the two groups. The size of the type 2 error is 23.6% and 21.7%.

## Discussion

In representative samples of individuals from two large cohort studies with visual acuity above the legal limit to drive,^6^ adults born preterm with VLBW were less likely to hold a driver’s licence than those born at term. This is in agreement with earlier reports where fewer adults born preterm with VLBW drove a car than term-born peers^2 3^ and is an interesting finding because foregoing driver’s licensure may be a real-life outcome of a person’s ability to perform complex tasks. The impediments to acquiring a driver’s licence may be numerous, but the coexistence with being born VLBW and lower BCVA may indicate that brain function is involved. Previous studies have linked VLBW with lower cognitive abilities^4^ and difficulties with visual perception,^9 10^ and a recent study established the link between mood disorders and no driver’s licence in the general population.^11^ In the present study, participants fulfilled the visual legal requirements and were thus not restrained by poor vision to acquire a driver’s licence. Earlier studies have identified various causes among young people, citing costs as well as health difficulties as the main reasons.^12^ However, driving a vehicle requires an ability to function in complex situations that may be difficult to attain for a proportion of adults born preterm.^4^ The obstacles to obtaining a driver’s licence may be linked to factors that are also associated with being born preterm with VLBW. A potential limitation of the study is that participants were not asked about the reason why they had no driver’s licence. Moreover, CIs were wide due to the relatively small sample size. Thus, non-significant findings should be interpreted with caution and there is a non-negligible statistical uncertainty in the findings.

## Conclusion

In a population with good visual acuity, being born preterm with VLBW was a predictor for not having a driver’s licence, while lower visual acuity further increased this likelihood. A history of a somatic or psychiatric diagnosis did not appear to contribute to this association in midlife, although this finding should be interpreted with caution. The causal relationship between being born preterm and not holding a driver’s licence remains to be investigated.

## Data Availability

Data are available on reasonable request.
